# Outcome and Structural Integrity of Rotator Cuff after Arthroscopic Treatment of Large and Massive Tears with Double Row Technique: A 2-Year Followup

**DOI:** 10.1155/2013/914148

**Published:** 2013-02-28

**Authors:** Ignacio Carbonel, Angel A. Martínez, Elisa Aldea, Jorge Ripalda, Antonio Herrera

**Affiliations:** ^1^Shoulder and Elbow Unit, Department of Orthopaedic and Trauma Surgery, Miguel Servet University Hospital, Zaragoza, Spain; ^2^Emergency Department, Royo Villanova Hospital, Zaragoza, Spain; ^3^Department of Orthopaedic and Trauma Surgery, Miguel Servet University Hospital and Department of Orthopaedic Surgery, School of Medicine, University of Zaragoza, Zaragoza, Spain

## Abstract

*Purpose*. The purpose of this study was to evaluate the functional outcome and the tendon healing after arthroscopic double row rotator cuff repair of large and massive rotator cuff tears. *Methods*. 82 patients with a full-thickness large and massive rotator cuff tear underwent arthroscopic repair with double row technique. Results were evaluated by use of the UCLA, ASES, and Constant questionnaires, the Shoulder Strength Index (SSI), and range of motion. Follow-up time was 2 years. Magnetic resonance imaging (MRI) studies were performed on each shoulder preoperatively and 2 years after repair. *Results*. 100% of the patients were followed up. UCLA, ASES, and Constant questionnaires showed significant improvement compared with preoperatively (*P* < 0.001). Range of motion and SSI in flexion, abduction, and internal and external rotation also showed significant improvement (*P* < 0.001). MRI studies showed 24 cases of tear after repair (29%). Only 8 cases were a full-thickness tear. *Conclusions*. At two years of followup, in large and massive rotator cuff tears, an arthroscopic double row rotator cuff repair technique produces an excellent functional outcome and structural integrity.

## 1. Introduction

Maximizing tendon healing is the primary goal of rotator cuff repair surgery. Tendon healing has been shown to improve active motion, strength, and patient self-assessed function after rotator cuff repair [1, 2].

Whereas small and medium sized rotator cuff tears are successfully managed with any surgical repair in the vast majority of cases, the optimal management of big and massive rotator cuff tears has been controversial and continues to evolve. Tendon retraction, adhesions, and poor tissue quality common in these tears make repair one of the most technically complex procedures in the shoulder.

Arthroscopic techniques and instrumentations are improving rapidly, and arthroscopic rotator cuff repair has gained popularity. Double row of anchors technique is reported to reestablish the normal rotator cuff footprint and increase the contact area for healing [3, 4] so anatomical and biomechanical are better than with the single row technique [4–8].

The purpose of this study was to evaluate the functional outcome and the tendon healing after arthroscopic double row rotator cuff repair of large and massive rotator cuff tears.

## 2. Materials and Methods

### 2.1. Patient Selection

Patients were recruited among those referred by primary care doctors because of symptoms of rotator cuff tears and were enrolled in the study by the senior surgeons of the Shoulder and Elbow unit at the Miguel Servet University Hospital in Zaragoza, Spain.

Recruitment started in January 2008 and was completed in January 2010. 82 patients were eligible. All patients received the allocated treatment.

Of 82 patients recruited, 2-year results were available for all of them.

Inclusion criteria included the following: (1) rotator cuff tear, clinically confirmed, in a sane patient with complete passive range of motion with inability to perform activities of daily living, (2) full-thickness tears bigger than 30 mm with MRI evidence, (3) older than 18, (4) no tendinous surgery in the shoulder, (5) healthy contralateral shoulder, and (6) informed consent for the surgery.

Exclusion criteria included the following: (1) glenohumeral osteoarthritis, (2) ipsilateral shoulder pathology, (3) previous rotator cuff surgery on the affected shoulder, (4) contralateral shoulder pathology, (5) fatty degeneration grade 4 of Fuchs, (7) the active use of steroids, and (8) inability to complete questionnaires or to complete the rehabilitation treatment.

### 2.2. Clinical Evaluation

Preoperative and postoperative clinical evaluations were performed on all patients preoperatively and 2 years postoperatively. Data were collected to allow a determination of the University of California, Los Angeles (UCLA) score [9], Constant-Murley score [10], and the shoulder index of the American Shoulder and Elbow Surgeons (ASES) [11]. The range of motion was evaluated with a goniometer in flexion, abduction, internal rotation, and external rotation. Muscle strength was tested using a spring-scale myometer (Manley 2012 spring-scale; Manley Tool and Machine, Independence, MO). Flexion, abduction, internal rotation, and external rotation muscle power were evaluated. We compared muscle strength using a new evaluation method, the Shoulder Strength Index (SSI). Instead of using the absolute value of the muscle strength, we used relative muscle strength of the affected shoulder compared with the muscle strength of the contralateral shoulder. Because normal muscle strength for each patient is totally different from that of others, comparison of the absolute values is meaningless. To calculate the SSI, muscle strength of the affected shoulder is divided by the muscle strength of the contralateral shoulder. The strength of the muscle power of both shoulders should be evaluated consecutively to ensure reproducibility and reliability.

### 2.3. Imaging

All patients received a standard preoperative assessment using standard radiographs: anteroposterior projections; neutral, external, and internal rotation; and an axillary view.

Nonarthrographic MRI studies were performed on all patients preoperatively and 2 years after repair. This has been shown to be a reliable method of evaluating the repaired rotator cuff [12–14]. The following sequences were performed: coronal and sagittal T1-weighted with TR 500/TE 15, coronal and sagittal FSE T2-weighted with fat saturation 4500/60, and axial FSE proton density with fat saturation 2500/12.

All scans were read by a musculoskeletal specialty. Size of the tear in the anteroposterior dimension, retraction of the tendon medially, and fatty degeneration were recorded in preoperative scans. Postoperative scans divided the rotator cuff in three groups: (1) full-thickness tear, defined as fluid signal and/or absence of visible tendon fibers extending across the entire tendon from inferior to superior, (2) partial-thickness tear defined as a partial tendon defect, and (3) cuff integrity when appeared to have sufficient thickness compared with normal cuff with homogenously low intensity on each image.

### 2.4. Surgical Technique

 All the operations were performed in a standardized manner. Patients underwent brachial plexus block associated with general anesthesia and were placed in a lateral decubitus position. The arm was suspended at approximately 30°–45° of abduction and 20° of forward flexion. Distraction of the shoulder joint was accomplished with 4 kg of traction. To control bleeding we used radiofrequency, adrenalin admixture for the irrigation fluid and asked the anesthesiologist to lower the systolic blood pressure to 90 mm Hg if possible. We worked with an arthroscopic pump maintained fluid pressure at 50 mm Hg, increasing it temporally on demand up to 75 mm Hg.

Standard anterior and posterior portals were produced, and the arthroscope was inserted into the glenohumeral joint through the posterior portal. A diagnostic arthroscopy was then performed to evaluate the extent of the rotator cuff tear, any lesions of the biceps tendon, and other associated lesions. The arthroscope was then removed from the glenohumeral joint and redirected into the subacromial space. After complete bursectomy was performed, arthroscopic subacromial decompression was performed to create a flat acromial undersurface in all patients. Osteophytes in the inferior part of the acromioclavicular joint were also removed, because not only an anterolateral subacromial spur but also medial subacromial spur and inferior clavicular spur were suspected as a cause of subacromial impingement. Tear size, pattern, and mobility of the torn cuff were evaluated. The edges of the tendon were debrided until strong healthy tissues were secured. For reattachment of the rotator cuff tendons, a cancellous bone bed was prepared in the footprint of the greater tuberosity with a bur until bleeding occurred. If mobility of a tendon was insufficient for repair, procedures to mobilize the tendon, such as release of the coracohumeral ligament and detachment of the rotator cuff from the bursal and articular sides, were performed.

The standard operating portals included the lateral portal for instrumentation, an accessory superior portal for anchor placement, and the previously established anterior and posterior portals. Frequently anterolateral and posterolateral portals are used.

The anchors used were Bio-corkscrew double loaded with number 2 FiberWire sutures (Arthrex, Naples, FL). These anchors were used by the 3 surgeons in both techniques.

For double row repairs, 1 row of anchors was placed in the medial aspect of the footprint, just lateral to the articular surface of the humeral head. Both sutures were passed through the tendon in a mattress fashion. A lateral row of anchors was then placed in the lateral aspect of the footprint, slightly proximal to the greater tuberosity. The lateral row sutures were passed in a simple suture fashion. Just 1 of the 2 sutures was passed through the tendon. When sutures had been placed, they were sequentially tied using a locking, sliding knot with back-up half-hitches. In all cases it was able to reestablish the anatomical footprint.

The L-shaped and U-shaped tears were first repaired with a side-to-side suture, providing margin convergence of the 2 edges of the cuff, before the fixation of the cuff to the bone.

### 2.5. Postoperative Management

The postoperative protocol was the same for the 82 cases. The arm was supported using a sling with an abduction pillow. Active elbow and wrist flexion and extension movements and pendular movements of the shoulder were initiated on the day after surgery. After 3 weeks, passive and assisted-active exercises were initiating. At 6 weeks, the sling was removed and the patient began active movements and strengthening exercises of the rotator cuff and scapular stabilizers progressively. Rehabilitation was consistently performed with the assistance of physical therapists. Full return to sports and heavy labor were allowed after 6 months according to individual functional recovery.

### 2.6. Statistical Methods

A statistical consultant recommended comparative tests based on the distribution of the data. Prerepair and postrepair comparisons were made using paired *t*-tests when data were normally distributed. For data that were not normally distributed, the Mann Whitney rank sum test was used. The significance level was set at *P* = 0.05.

All statistical analyses were performed by an independent statistician using the SPSS statistical package version 11.0.

## 3. Results

This study includes 82 patients, 36 males and 46 females. The mean ages of patients were 58.33 ± 5.2. The mean area of rotator cuff tear was 43 ± 6.1 mm^2^.

The mean number of anchors used in double row repair technique was 3.28 ± 0.6 (Table 1).

### 3.1. Patient Functional Assessment

UCLA postoperative score was 27.6 ± 1.7; ASES index was 82.7 ± 3.1; and Constant score was 76.1 ± 2.3. The scores showed a significant improvement compared with preoperative status (*P* < 0.001) (Table 2).

### 3.2. Range of Motion

Patients' active flexion increased significantly from 92° to 145° (*P* < 0.001). Active abduction increased significantly from 90° to 125° (*P* < 0.001). Active external rotation increased significantly from 44° to 56° (*P* < 0.001). Active internal rotation increased significantly from 40° to 52° (*P* < 0.001) (Table 3).

### 3.3. Strength Measurements: SSI

All the measurements of strength (flexion, abduction, and internal and external rotation) showed a significant improvement in postoperatively results (Table 4).

### 3.4. Structural Integrity by MRI

MRI studies at final followup showed an intact rotator cuff in 58 patients, partial-thickness defects in 16 patients, and full-thickness defects in 8 patients (Table 5).

Range of motion and patient functional assessment showed difference between cases of intact repair and retear (Table 6).

## 4. Discussion

Recent arthroscopic repair techniques for rotator cuff tears have emphasized the potential for a double row repair to add strength to the repair and hopefully decrease the anatomic failure rate [15–20]. Several studies have indicated that results in cases of anatomic failure, although clinically improved, are not as good as those that are anatomically intact, especially if strength measurements are made [15, 21, 22]. Therefore, trying to achieve and maintain an intact cuff is a paramount goal in cuff repair. Biomechanical studies have emphasized the potential improvement of outcomes by double row repair technique [23–28]. Some clinical studies have already validated this idea in large and massive rotator cuff tears [29].

Park et al. [29] recently conducted a study in which 40 consecutive patients were treated with 1 row technique and the following 38 with double row technique. At 2 years after surgery, ASES, Constant, and SSI were significantly better in both groups but no significant improvements were found between both groups. When the comparison was made to the size of the rupture, functional assessment was significantly better with the double row in large and massive tears (>3 cm) (*P* < 0.05). The authors concluded that the repair with the double row technique has a greater role in the treatment of large and massive tears.

Also Denard et al. [30] concluded that double row repair was 4.9 times more likely to lead to a good or excellent long-term functional outcome after repair of massive rotator cuff tears.

Lafosse et al. [18] evaluated 105 patients undergoing arthroscopic double row repair of rotator cuff tears, enjoying positive results functionally and structurally. The improvement in the Constant scale was 43.2 ± 15.1 preoperatively to 80.1 ± 11.1 after a mean of 23 months. Assessment by MRI and arthroTC revealed a total of 12 failures (11%), a lower rate than previously collected with other techniques of repair with tears comparable in size and shape. In shoulders with an intact cuff at followup was also observed a better clinical outcome. The only category in which they found a statistically significant difference was the assessment of pain (*P* < 0.0001). 

Anderson et al. [15] published a series of 48 patients (52 shoulders) who underwent doublerow repair in full thickness rotator cuff tears. The average size of the tear was of 2.47 cm. Postoperative followup included functional assessment, physical examination, ultrasound, and tests of strength. With a mean followup of 30 months, ultrasound showed a 17% of tears. The L'Insalata scale (*P* < 0.001), mobility (*P* < 0.001), and strength (antepulsion, *P* < 0.001, external rotation, *P* < 0.001; internal rotation, *P* < 0.033) were statistically significant improvements with respect to preoperative values. Patients with an intact rotator cuff showed a significant improvement in antepulsion (*P* = 0.006) and external rotation (*P* = 0.001). 

Huijsmans et al. [17] presented the results of 238 patients undergoing repair with a new doublerow suture technique. 90% of patients had a performance status of good to excellent, and 82.9% remained intact after the repair with ultrasound control. 

A new evaluation method, the SSI, only used in Park et al.'s article [29], was introduced and reflects patients subjective judgement about the operation as well as their rehabilitation status. Because patients will always compare the operated shoulder with the unaffected shoulder, the only standard function of the shoulder will be that of the unaffected shoulder. Using the SSI, clinicians can more easily explain the goals of surgery and rehabilitation to the patient. In addition, data from the SSI indicate that arthroscopic rotator cuff repair will restore about 80% of muscle power to the unaffected shoulder. These data could be used to counsel patients before surgery.

Follow-up time is also an important aspect for evaluation of these results but it is a fact that there is no clear consensus. Some studies say that the failures appear late in large and massive tears [3], while in the open repair technique, improvements appear overtime. In other studies, these failures appear in very early stages after repair [31] while others appear in later stages of evolution [32], In our study we followed up for 24 months because we have observed that it is long enough to assess the functional recovery of the shoulder and problems with the surgery appear early after surgery.

The failure rate in most of the other studies is around 15–20% [19, 29, 31, 33]. Our results are higher (29%) but our study only included large and massive tears. There are few studies that showed results in large and massive tears and results are variable, from 17% to 44% retear rates [17–20].

An area of inconsistency within the literature is whether the presence of a defect after rotator cuff repair necessarily affects functional outcome. In our study we detected differences in strength in full thickness defects but in partial thickness defects these differences were less significative. Klepps et al. [34], as well as Knudsen et al. [35], detected no significant effect of repair integrity on functional outcome. Harryman et al. [2] had similar results except with those who had a large recurrent defect. However, Gazielly et al. [1] found a close correlation between the functional score and the anatomical condition of the rotator cuff.

Radiologic method of pre- and postoperative evaluation may also account for some of the variability of failure results. We used noncontrast magnetic resonance imaging to evaluate postoperative rotator cuff repair integrity. It is approved as a proper imaging test for this purpose and the most commonly used in these studies but it has somewhat limited ability in differentiating scar tissue from healed rotator cuff. High resolution shoulder ultrasonography has been evaluated and found to have an extremely high sensitivity (91%), specificity (86%), and accuracy (89%) in correctly identifying rotator cuff integrity postoperative [36]. Ultrasonography also provides dynamic evaluation of the rotator cuff, improving the ability to differentiate scar from healed cuff, although the results are highly operator dependent, and at our institution there are no musculoskeletal radiologists with extensive ultrasound experience and we achieve better results with noncontrast magnetic resonance imaging.

## 5. Conclusion

At two years of follow-up, in large and massive rotator cuff tears, an arthroscopic double row rotator cuff repair technique produces an excellent functional outcome and structural integrity.

## Figures and Tables

**Table 1:** Tear description.

Mean age (years)	58.33 ± 5.2
Gender	
Male	36
Female	46
Mean area (mm^2^)	43 ± 6.1
Number of anchors	3.28 ± 0.6

**Table 2:** Functional assessment.

	Media ± DS		*P *
	Preop	Postop
UCLA (0–35)	11.6 ± 1.6	27.6 ± 1.7	<0.001
Constant (0–100)	42.2 ± 5.1	76.1 ± 2.3	<0.001
ASES (0–100)	41.3 ± 4.2	82.7 ± 3.1	<0.001

**Table 3:** Range of motion.

	Media ± DS		*P *
	Preop	Postop
External rotation, deg	44.2 ± 4.1	56.1 ± 3.2	<0.001
Internal rotation, deg	40.3 ± 4.3	52.8 ± 2.9	<0.001
Flexion, deg	92.7 ± 10.6	145.2 ± 6.7	<0.001
Abduction, deg	90.1 ± 9.8	125.6 ± 7.6	<0.001

**Table 4:** Strength measurements: SSI.

	Media ± DS		*P *
	Preop	Postop
Flexion SSI	0.50 ± 0.05	0.74 ± 0.03	<0.001
Abduction SSI	0.52 ± 0.06	0.72 ± 0.03	<0.001
Internal rotation SSI	0.61 ± 0.03	0.72 ± 0.03	<0.001
External rotation SSI	0.65 ± 0.03	0.78 ± 0.02	<0.001

**Table 5:** Imaging.

MRI	Intact	Partial-thickness defect	Full-thickness defect
Double row	58	16	8

**Table 6:** 

Rotator cuff repair	Intact	Partial-thickness defect	Full-thickness defect	*P *
External rotation, deg	61.3 ± 3.1	52.1 ± 2.9	45.2 ± 3.3	<0.001
Internal rotation, deg	58.1 ± 2.2	50.6 ± 3.5	39.5 ± 4.1	<0.001
Flexion, deg	151.3 ± 5.2	140.1 ± 4.7	115.8 ± 7.2	<0.001
Abduction, deg	135.4 ± 4.7	120.5 ± 5.2	102.3 ± 5.2	<0.001
UCLA	28.9 ± 2.1	26.8 ± 1.9	24.7 ± 2.5	<0.001
Constant	80.3 ± 3.9	74.1 ± 4.3	63.2 ± 4.7	<0.001
ASES	85.0 ± 4.3	79.7 ± 5.1	71.9 ± 4.1	<0.001

## References

[1] Gazielly DF, Gleyze P, Montagnon C (1994). Functional and anatomical results after rotator cuff repair. *Clinical Orthopaedics and Related Research*.

[2] Harryman DT, Mack LA, Wang KY, Jackins SE, Richardson ML, Matsen FA (1991). Repairs of the rotator cuff: correlation of functional results with integrity of the cuff. *Journal of Bone and Joint Surgery A*.

[3] Galatz LM, Ball CM, Teefey SA, Middleton WD, Yamaguchi K (2004). The outcome and repair integrity of completely arthroscopically repaired large and massive rotator cuff tears. *Journal of Bone and Joint Surgery A*.

[4] Lo IKY, Burkhart SS (2003). Double-row arthroscopic rotator cuff repair: re-establishing the footprint of the rotator cuff. *Arthroscopy*.

[5] Brady PC, Arrigoni P, Burkhart SS (2006). Evaluation of residual rotator cuff defects after in vivo single- versus double-row rotator cuff repairs. *Arthroscopy*.

[6] Curtis AS, Burbank KM, Tierney JJ, Scheller AD, Cunan AR (2006). The insertional footprint of the rotator cuff: an anatomic study. *Arthroscopy*.

[7] Park MC, ElAttrache NS, Tibone JE, Ahmad CS, Jun BJ, Lee TQ (2007). Part I: footprint contact characteristics for a transosseous-equivalent rotator cuff repair technique compared with a double-row repair technique. *Journal of Shoulder and Elbow Surgery*.

[8] Waltrip RL, Zheng N, Dugas JR, Andrews JR (2003). Rotator cuff repair: a biomechanical comparison of three techniques. *American Journal of Sports Medicine*.

[9] Ellman H, Hanker G, Bayer M (1986). Repair of the rotator cuff: end-result study of factors influencing reconstruction. *Journal of Bone and Joint Surgery A*.

[10] Constant CR, Murley AHG (1987). A clinical method of functional assessment of the shoulder. *Clinical Orthopaedics and Related Research*.

[11] Richards RR, An KN, Bigliani LU (1994). A standardized method for the assessment of shoulder function. *Journal of Shoulder and Elbow Surgery*.

[12] Fuchs B, Gilbart MK, Hodler J, Gerber C (2006). Clinical and structural results of open repair of an isolated one-tendon tear of the rotator cuff. *Journal of Bone and Joint Surgery A*.

[13] Jost B, Pfirrmann CWA, Gerber C, Switzerland Z (2000). Clinical outcome after structural failure of rotator cuff repairs. *Journal of Bone and Joint Surgery A*.

[14] Teefey SA, Rubin DA, Middleton WD, Hildebolt CF, Leibold RA, Yamaguchi K (2004). Detection and quantification of rotator cuff tears: comparison of ultrasonographic, magnetic resonance imaging, and arthroscopic findings in seventy-one consecutive cases. *Journal of Bone and Joint Surgery A*.

[15] Anderson K, Boothby M, Aschenbrener D, Van Holsbeeck M (2006). Outcome and structural integrity after arthroscopic rotator cuff repair using 2 rows of fixation: minimum 2-year follow-up. *American Journal of Sports Medicine*.

[16] Apreleva M, Özbaydar M, Fitzgibbons PG, Warner JJP (2002). Rotator cuff tears: the effect of the reconstruction method on three-dimensional repair site area. *Arthroscopy*.

[17] Huijsmans PE, Pritchard MP, Berghs BM, Van Rooyen KS, Wallace AL, De Beer JF (2007). Arthroscopic rotator cuff repair with double-row fixation. *Journal of Bone and Joint Surgery A*.

[18] Lafosse L, Brozska R, Toussaint B, Gobezie R (2007). The outcome and structural integrity of arthroscopic rotator cuff repair with use of the double-row suture anchor technique. *Journal of Bone and Joint Surgery A*.

[19] Sugaya H, Maeda K, Matsuki K, Moriishi J (2007). Repair integrity and functional outcome after arthroscopic double-row rotator cuff repair: a prospective outcome study. *Journal of Bone and Joint Surgery A*.

[20] Sugaya H, Maeda K, Matsuki K, Moriishi J (2005). Functional and structural outcome after arthroscopic full-thickness rotator cuff repair: single-row versus dual-row fixation. *Arthroscopy*.

[21] Bishop J, Klepps S, Lo IK, Bird J, Gladstone JN, Flatow EL (2006). Cuff integrity after arthroscopic versus open rotator cuff repair: a prospective study. *Journal of Shoulder and Elbow Surgery*.

[22] Boileau P, Brassart N, Watkinson DJ, Carles M, Hatzidakis AM, Krishnan SG (2005). Arthroscopic repair of full-thickness tears of the supraspinatus: does the tendon really heal?. *Journal of Bone and Joint Surgery A*.

[23] Kim DH, ElAttrache NS, Tibone JE (2006). Biomechanical comparison of a single-row versus double-row suture anchor technique for rotator cuff repair. *American Journal of Sports Medicine*.

[24] Ma CB, Comerford L, Wilson J, Puttlitz CM (2006). Biomechanical evaluation of arthroscopic rotator cuff repairs: double-row compared with single-row fixation. *Journal of Bone and Joint Surgery A*.

[25] Meier SW, Meier JD (2006). The effect of double-row fixation on initial repair strength in rotator cuff repair: A Biomechanical Study. *Arthroscopy*.

[26] Meier SW, Meier JD (2006). Rotator cuff repair: the effect of double-row fixation on three-dimensional repair site. *Journal of Shoulder and Elbow Surgery*.

[27] Smith CD, Alexander S, Hill AM (2006). A biomechanical comparison of single and double-row fixation in arthroscopic rotator cuff repair. *Journal of Bone and Joint Surgery A*.

[28] Tuoheti Y, Itoi E, Yamamoto N (2005). Contact area, contact pressure, and pressure patterns of the tendon-bone interface after rotator cuff repair. *American Journal of Sports Medicine*.

[29] Park JY, Lhee SH, Choi JH, Park HK, Yu JW, Seo JB (2008). Comparison of the clinical outcomes of single- and double-row repairs in rotator cuff tears. *American Journal of Sports Medicine*.

[30] Denard PJ, Jiwani AZ, Ladermann A, Burkhart SS (2012). Long-term outcome of arthroscopic massive rotator cuff repair: the importance of double-row fixation. *Arthroscopy*.

[31] Gerber C, Fuchs B, Hodler J (2000). The results of repair of massive tears of the rotator cuff. *Journal of Bone and Joint Surgery A*.

[32] Burks RT, Crim J, Brown N, Fink B, Greis PE (2009). A prospective randomized clinical trial comparing arthroscopic single- and double-row rotator cuff repair: magnetic resonance imaging and early clinical evaluation. *American Journal of Sports Medicine*.

[33] Grasso A, Milano G, Salvatore M, Falcone G, Deriu L, Fabbriciani C (2009). Single-row versus double-row arthroscopic rotator cuff repair: A Prospective Randomized Clinical Study. *Arthroscopy*.

[34] Klepps S, Bishop J, Lin J (2004). Prospective evaluation of the effect of rotator cuff integrity on the outcome of open rotator cuff repairs. *American Journal of Sports Medicine*.

[35] Knudsen HB, Gelineck J, Søjbjerg JO, Olsen BS, Johannsen HV, Sneppen O (1999). Functional and magnetic resonance imaging evaluation after single-tendon rotator cuff reconstruction. *Journal of Shoulder and Elbow Surgery*.

[36] Prickett WD, Teefey SA, Galatz LM, Calfee RP, Middleton WD, Yamaguchi K (2003). Accuracy of ultrasound imaging of the rotator cuff in shoulders that are painful postoperatively. *Journal of Bone and Joint Surgery A*.

